# Targeting Langerhans cells via skin delivery of HIV Envelope enhances the antibody response to vaccination

**DOI:** 10.1038/s41541-025-01214-w

**Published:** 2025-07-25

**Authors:** Juliane S. Lanza, Adele Hammoudi, Joanna De Chiara, Mathieu Surenaud, Anaïs Kembou, Michela Esposito, Sandra Zurawski, Gerard Zurawski, Mireille Centlivre, Bernard Malissen, Véronique Godot, Yves Lévy, Sandrine Henri, Sylvain Cardinaud

**Affiliations:** 1 Vaccine Research Institute (VRI), INSERM-U955 (IMRB) Équipe 16, Université Paris-Est Créteil (UPEC), Créteil, France; 2 Centre d’Immunologie de Marseille-Luminy (CIML), Aix Marseille Université, INSERM, CNRS, Parc Scientifique et Technologique de Luminy, Marseille, France; 3 Centre d’immunophénomique (CIPHE), Aix Marseille Université, INSERM, CNRS, Parc Scientifique et Technologique de Luminy, Marseille, France; 4 Baylor Institute for Immunology Research (BIIR), Dallas, TX USA; 5 Assistance Publique-Hôpitaux de Paris, Groupe Henri-Mondor Albert-Chenevier, Service Immunologie Clinique, Créteil, France

**Keywords:** Protein vaccines, Germinal centres, Immunological memory, Antibodies, HIV infections

## Abstract

Targeting dendritic cells (DCs) with antigens is a promising approach to modulating T follicular helper (Tfh) cells and germinal center (GC) reactions, enhancing vaccine-induced adaptive immune responses, with preclinical studies highlighting a key role of Langerhans cells (LCs) in generating HIV-1-specific antibody responses. This study evaluated the immunogenicity of a Langerin-targeting vaccine (αLang.Env), comprising an anti-mouse Langerin mAb fused to HIV-1 Envelope 96ZM651 gp140 (Env), delivered through various skin immunization routes in mice, and explored the roles of epidermal LCs and dermal cDC1s in adaptive immune responses. Lymph nodes draining the immunization sites were analyzed using ovalbumin (OVA) as a surrogate antigen after topical (top.), subcutaneous (s.c.), intradermal (i.d.), or transcutaneous (t.c.) delivery via laser-guided microporation, with αLang.Env administered without adjuvant in a Prime-Boost scheme. All methods primed T cells in draining lymph nodes (dLN), as shown by OVA-specific CD8^+^ and CD4^+^ T cell proliferation, while αLang.Env induced GC B cells regardless of the route. However, topical delivery did not elicit Tfh cells or Env-specific GC B cells, whereas i.d. and s.c. routes produced systemic Env-specific IgG responses, with i.d. immunization yielding the highest titers and strongest Tfh responses. In the *Xcr1*^DTA^ mouse model, where cDC1s were depleted, the i.d. route confirmed that epidermal LCs were the primary drivers of GC/Tfh reactions and humoral responses, while cDC1s mediated CD8^+^ T cell effector responses. These findings highlight that i.d. administration of the HIV-1 Env antigen targeted to Langerin, without the use of an adjuvant, is an effective vaccine strategy for eliciting GC reactions in LN and generating robust antibody responses, primarily through the activation of LC.

## Introduction

The effectiveness of vaccination strategies depends on their capacity to trigger strong and long-lasting immune responses, primarily mediated by T and B lymphocytes. Ideally, a vaccine should induce lifelong, complete immunity. However, very few vaccines achieve such a high level of protection. In the case of HIV-1, there is still no efficient vaccine available. The RV-144 trial, utilizing a recombinant canarypox vector prime vaccine followed by a boost with a recombinant HIV-1 surface protein Env subunit (HIV-1 glycoprotein 120, gp120), showed limited protection against HIV-1 infection that was associated with the generation of anti-Env antibodies binding V1V2 viral regions^1^. However, these responses diminished over time, highlighting that the vaccine failed to induce enduring V1V2 antibodies. Since then, these findings were not replicated in various following phase 2b/3 HIV-1 prophylactic studies^2,3^. These subsequent studies tested different immunogens and/or adjuvants pointing out that the choice of antigen, adjuvants, and administration routes are all crucial elements in vaccine design and influence immune responses and vaccine efficacy^4^. These observations are in line with recent advances in understanding of induction and regulation of the immune response to generate a combined T and B cell memory against complex antigens^5^.

Among several strategies for protection against HIV-1 infection, a promising rational approach involves targeting antigens to endogenous dendritic cells (DCs) to enhance their presentation to naïve lymphocytes and favor the generation of memory T and B cells as part of adaptive immunity^6,7^. The conventional approach to develop vaccines targeting DCs is to use a specific monoclonal antibody (mAb) directed against a particular endocytic DC receptor^8^. DCs are specialized antigen-presenting cells (APCs) crucial for the integration of innate and adaptive immune response. DCs carry, process, and present antigens to T lymphocytes in lymphoid organs, thus initiating and controlling the early steps of the adaptive immune response^9^. Vaccine-activated DCs together with helper CD4^+^ T cells can further activate naïve T and B cells generating either memory CD8^+^ cytolytic T cells, that will be able to kill infected cells, or inducing plasma B cells that further mature within the structured GC of the dLN, accompanied by the secretion of antigen-binding IgG, showing neutralizing or non-neutralizing antiviral functions^10–13^. These processes can be deemed predictive and indicative of immunity against pathogens^14–17^. But DC-mediated regulation of host immune responses is a complex coordination of events and has mainly been explained by the functional plasticity of DCs driven by external signals as well as the presence and location of different subsets of DCs. Understanding each phenotype, specific markers and its regulation upon activation, location and migration is essential to design improved DC-targeting vaccines.

The skin, one of the largest barriers to pathogen entry as well as a site for vaccine antigen administration, contains diverse immune cells. It is divided into three layers: the epidermis, the dermis and the hypodermis. The dermis contains blood and lymph vessels that facilitate the recruitment of blood-borne cells and the migration of activated dermis-resident cells towards the draining lymph nodes (dLN), respectively^18^. Extravasated blood Ly6C^hi^ monocytes generate dermal monocyte-derived DC and dermal monocyte-derived macrophages which reside in the dermis and the latter are probably derived from both embryonic progenitors and blood Ly6C^hi^ monocytes. In mice, the conventional dendritic cells (cDCs) reside in the dermis and develop from blood-derived pre-cDC precursors^19–21^, and together with Langerhans cells (LCs) that reside in the epidermis and originate from yolk sac-derived progenitors, cDCs are endowed with the capacity to migrate to the dLNs. The phenotype and function of cell subpopulations, in particular epidermal LCs and dermal DCs, have been essentially described in mouse models^22–25^. The expression of XC-chemokine receptor 1 (XCR1) robustly defines the unique cDC1 subset across tissues and species^26^, also known as CD103^+^, CD8α^+^ DCs and these also express CD207 in mice, like LCs. Dermal cDC1 is endowed with the unique capacity to cross-present antigens expressed by keratinocytes^22^. This observation challenged the initial dogma stipulating that LCs would be the exclusive APCs of the skin. LCs exhibit high expression of the C-type lectin Langerin (CD207, Clec4K), which binds strongly to mannose residues. The mannose-CD207-bound complexes are internalized into Birbeck granules, facilitating access to the non-classical antigen processing and presentation pathway^27^. Skin LCs appear to maintain epidermal homeostasis and tolerance to commensals, while retaining the ability to respond to selected intracellular pathogens and viruses under inflammatory conditions. In skin, LCs can mature into potent cross-presenting APCs. Some works indicated that LCs preferentially select and expand antigen-specific CTLs, and induce high avidity CTLs^28–30^. LCs are also potent inducers of Th1-, Th2-, Th17 and Th22-type CD4^+^ T cell responses^31,32^. Additionally, targeting LCs in vivo and in vitro also revealed a critical role in priming Tfh and GC B cell responses^33–37^. Despite this extensive research over the past years, it remains difficult to determine the specific conducive role of cDC and/or LCs in initiating Tfh cell differentiation, GC reactions in LN, and effective cellular and humoral responses. Particularly in vaccinology, investigating the contribution of each subset to immunity is challenging due to the complex coordination between cDCs, LCs, T, and B cells. Hence, the development of effective immunization strategies, schemes and routes of immunization relies on a comprehensive understanding of the initiation and regulation of humoral and cellular responses.

We previously demonstrated the anti-Langerin 4C7 mAb fused to the HIV-1-gp140z envelope (αLang.Env) promotes in vitro expansion of human Tfh cells and Env-specific B cells when cocultured with isolated LCs^34^. Additionally, this construct was shown to promote in vivo the GC/Tfh reaction in the dLN of the site of immunization and circulating Env-specific IgG were detected. Here, we aimed at deciphering the “in situ” targeting of HIV-1-gp140z (Env) to the Langerin receptor in different skin layers and after different schemes of skin vaccination in mice. To do so, we used Wild Type mouse controls and *Xcr1*^DTA^ mouse models (in a C57BL/6J background). Knock in *Xrc1*^DTA^ mice present a specific and conditional ablation of XCR1^+^ cells, meaning the specific absence of cDC1 in organs and tissues, including the skin^38^. Skin vaccination of *Xcr1*^DTA^ mice allows for the evaluation of the specific role of LCs, with Env antigen exclusively targeted to Langerin (αLang.Env) in generating GC reaction (Tfh cells, GC B and Env^+^ GC B cells) in proximal lymph nodes and Env-specific humoral response. On the other hand, *Xcr1*^Cre-mTFP1^ littermates and C57BL/6J mice were also used as controls for αLang.Env vaccination where anti-Langerin mAb will target both dermal/epidermal LC and cDC1s. With this, we aimed to evaluate the role of LCs and cDC1s in the generation of the early events of the immunity, i.e., Tfh/GC reaction and humoral responses in proximal dLNs and blood, as well as the influence of skin routes of immunization in the immunogenicity of αLang.Env vaccine candidate.

## Results

### Following OVA antigen immunization, all routes of immunizations induced OT-I and OT-II T cell proliferation in specific dLNs

**Fig. 1:**
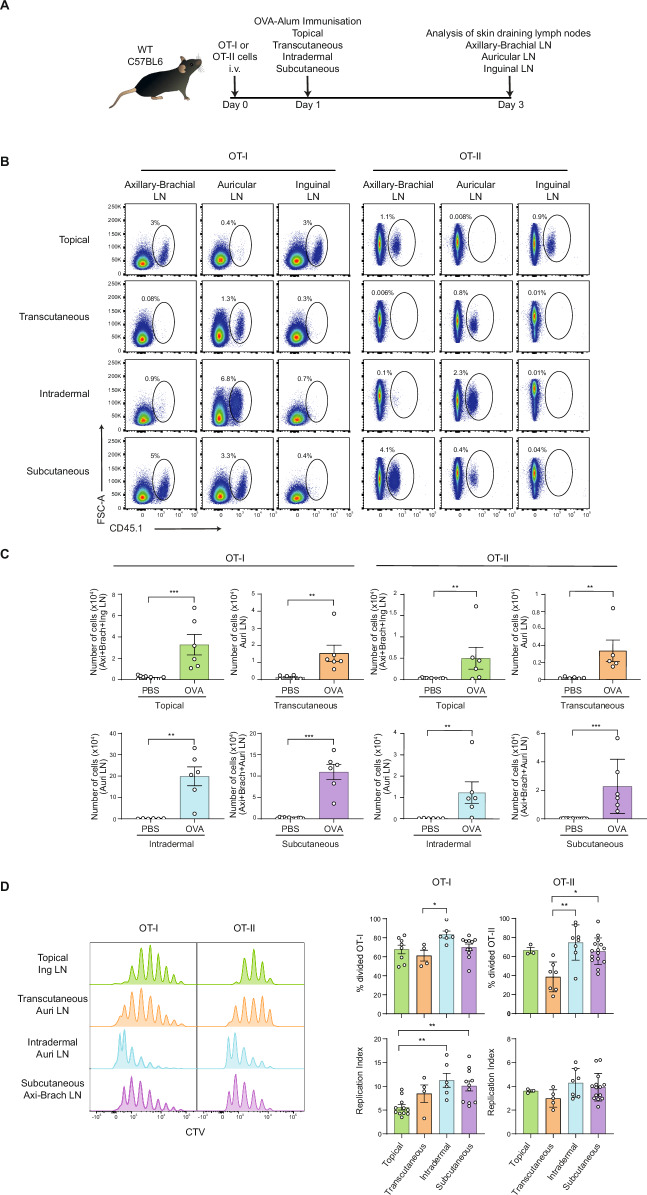
OVA-specific CD8^+^ and CD4^+^ T cell activation and proliferation in distinct proximal lymph nodes depends on immunization site. A surrogate vaccine formulation was used to test the potential of different skin routes of immunization (topical, transcutaneous, intradermal and subcutaneous) to induce antigen-specific CD8^+^ T cell (OT-I) and CD4^+^ T cell (OT-II) proliferation in the dLN. **A** Experimental design – OT-I/OT-II T cell adoptive transfer and vaccination scheme. **B** FACS plots of CD45.1^+^ OT-I and CD45.1^+^ OT-II T cells in skin dLN (Axillary-Brachial; Auricular; Inguinal): CD45.1^+^ OT-I and CD45.1^+^ OT-II T cells were pre-gated as CD8^+^ Lin^-^ and CD4^+^ Lin^-^ respectively (See Sup Fig. 1). **C** Absolute number of CD45.1^+^ OT-I and CD45.1^+^ OT-II T cells in respective dLNs. Non-parametric Mann-Whitney unpaired t tests; **P < 0.01, ***P < 0.001. **D** (left) Proliferation profiles of CD45.1^+^ OT-I and CD45.1^+^ OT-II in respective dLNs (CTV peaks). (right) Division and replication indexes of CD45.1^+^ OT-I and CD45.1^+^ OT-II T cells in respective dLNs. Non-parametric Kruskal-Wallis tests with Dunn’s multiple comparison post hoc test, *P < 0.05, **P < 0.01. Non-significant responses are not indicated. Data are representative of at least two experiments.

Recognizing the pivotal role of immunization route in shaping immune outcomes, we first performed a comprehensive investigation to dissect the efficacy of different skin immunization modes on T cell priming. To increase the sensitivity and accuracy of cellular responses, we used the OVA-specific CD4^+^ (OT-II) and CD8^+^ (OT-I) T cells adoptive transfer and proliferation assay^39^. At day 0, CTV-labelled OT-I or OT-II T cells which express both allelic markers CD45.1 and CD45.2, were adoptively transferred to C57BL/6 mice (CD45.2). At day 1, the animals were immunized with OVA.CT.Alum formulation (10 µg OVA) via different routes of skin vaccination: subcutaneously (s.c.) or intradermally (i.d.) with syringe administration, transcutaneously (t.c.) via laser microporation, and topical application (top.) using a Hilltop chamber. Three days later, OT-I and OT-II T cell proliferation in auricular, brachial plus axillary, and inguinal lymph nodes was assessed by flow cytometry (Fig. 1A). By FACS, OT-I and OT-II T cells were gated respectively as CD3^+^CD8^+^CD45.1^+^ and CD3^+^CD4^+^CD45.1^+^ and based on the level of CTV we determined the proliferation induced by the immunizations (Supplementary Fig. S1A, B). We first compared the frequency of OT-I and OT-II T cells in axillary-brachial LN, auricular LN and inguinal LN for each route of immunization and found that following top. immunization, OT-I and OT-II T cells were detected in axillary-brachial and inguinal LNs, while after t.c. and i.d. immunization, OT-I and OT-II T-cells were detected only in auricular LN, and after s.c. immunization, OT-I and OT-II T cells were detected in axillary-brachial and auricular LNs, indicating the respective dLN for each of the route of immunization (Fig. 1B). Taking in account the different dLN depending on the route of immunization, the absolute numbers of OT-I and OT-II T cells were significantly increased compared to PBS-injected controls in all immunization routes (Fig. 1C). However, the potency of each immunization route in inducing OT-I and OT-II T cell proliferation was different. The percentages of OT-I and OT-II T cells were highest when immunizing through i.d. and s.c. routes, with mean fold changes of 34 and 39, respectively, compared to the PBS non-immunized group (Supplementary Fig. S1C, D). The i.d. route has shown to generate significantly higher absolute numbers of OT-I T cells (average fold change by 136), followed by s.c. (average fold change by 53), while t.c. and topical routes were lower (average fold change 15). The increase in OT-II T cell numbers post-immunization was higher with i.d. and s.c. administration (fold change of 58 and 74, respectively) compared to the t.c. and top. immunization routes (average fold increase of 18). Based on the percentage of divided cells or the replication index, i.d. and s.c. induced higher proliferation of OT-I and OT-II T cells (Fig. 1D). This suggests that the i.d. and s.c. routes are more efficient than the others tested for T cell priming. Altogether, this first approach using the surrogate OVA antigen showed that the four routes of immunization are followed by antigen presentation and induction of OT-I and OT-II T cell proliferation. i.d. and t.c. immunizations can be followed in the auricular LN, while s.c. immunization can be followed in the axillary-brachial and/or auricular LNs and we have chosen the axillary-brachial LNs for the rest of the study. Lastly, topical immunization can be followed in the inguinal and/or axillary-brachial LNs and for this purpose we have chosen the inguinal LN for the rest of the study.

### Intradermal immunization enhances αLang.Env vaccine immunogenicity

**Fig. 2:**
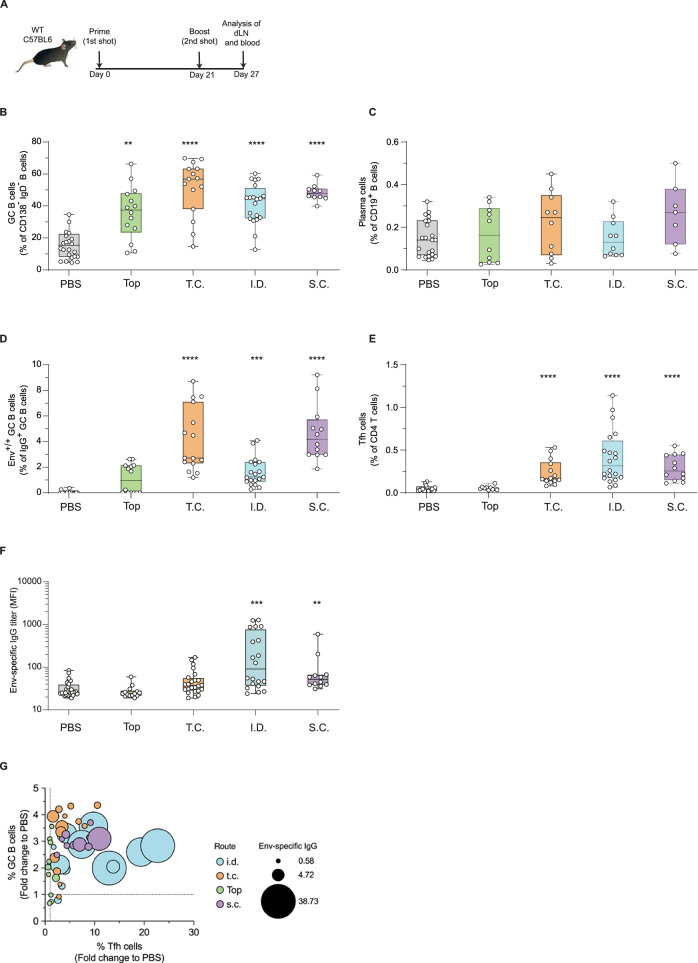
αLang.Env induces cellular and humoral responses in C57BL/6 mice via different routes of skin vaccination (topical, subcutaneous, intradermal, transcutaneous). **A** Prime-Boost vaccination scheme with 20 µg αLang.Env per mouse in 3 weeks interval. One week after the boost, blood and dLNs were harvested to analyze the cellular and humoral immune response. Frequencies of total GC B cells among CD138^–^ IgD^–^ CD19^+^ B cells (**B**), of plasma cells (**C**), of Env-specific GC B cells among IgG^+^ GC B cells (**D**), and of PD1^high^CXCR5^+^ Tfh cells among CD4^+^ T cells (**E**) in LNs draining each site of top (green), t.c. (orange), i.d. (cyan) and s.c. (purple) immunization. **F** Env-specific IgG levels in serum of mice, measured by Luminex® (reported as mean of fluorescent intensity, MFI) at D27. Non-parametric Kruskal-Wallis tests with Dunn’s multiple comparison post hoc test, *P < 0.05, **P < 0.01, ***P < 0.001, ****P < 0.0001. Non-significant responses are not indicated. Data are representative of at least two experiments. **G** For each immunization route, fold changes in Tfh and GC B cell responses are presented as ratios of frequencies compared to the PBS control group, while Env IgG titers are shown in comparison to the values of non-immunized mice (indicated by the size of the circles). Each circle represents an individual animal, with colors indicating the administration routes.

We and other collaborators previously showed that administration of recombinant anti-Langerin mAb through s.c. or intraperitoneal (i.p.) routes of immunization significantly improves GC/Tfh reaction and antigen-specific antibody responses^33,34,37^. We hypothesized that the targeting of Langerin in situ, using different skin routes of immunization could significantly influence the magnitude and quality of anti-HIV-1 humoral responses elicited by αLang.Env (vaccine structure and quality control depicted in Supplementary Figs. S2 and S6). To determine which route of skin immunization is more efficient to enhance the early immune events stimulated by non-adjuvanted Env-targeting Langerin to both LC and cDC1s, we chose a Prime-boost scheme in which αLang.Env was applied in different groups of C57BL/6 mice at the same dose (20 µg of vaccine) through s.c., i.d., t.c. and top. routes. The interval between each administration was 3 weeks and the response readout between 6-7 days after the boost (Fig. 2A).

Induction of Tfh and GC immune responses and Env-specific humoral response were evaluated by FACS immunophenotyping of dLN single cell suspensions and Luminex® assay in plasma. The Tfh cells were gated as CD5^+^CD3^+^CD4^+^PD1^high^CXCR5^+^ cells, plasma cells (PC) were gated as CD5^–^CD3^–^CD19^+^MHC-II^+^IgD^–^CD138^+^ B cells, while GC B cells were gated as CD5^–^CD3^–^CD19^+^MHC-II^+^IgD^–^CD138^–^GL7^+^CD95^+^ cells and among them, Env-specific IgG^+^ GC B cells were detected using biotinylated-Env protein and two anti-biotin fluorescent antibodies to increase the detection sensibility (Supplementary Fig. S3). In non-immunized mice, the frequencies of these populations were not significantly different in the LN draining each immunization site. The frequency of GC B cells in dLNs significantly increased after all tested immunization routes compared to the PBS-treated group (***P < 0.001) (Fig. 2B), though the absolute number of GC B cells remained unchanged following topical vaccine application (Supplementary Fig. S4A). While PC frequencies remained stable (Fig. 2C), their absolute numbers in dLNs significantly increased following t.c., i.d., and s.c. immunization routes (Supplementary Fig. S4B). Env-specific GC B cells were detectable, with significantly higher frequencies and absolute numbers following t.c., s.c. (*P* < 0.0001), and i.d. (*P* < 0.001) routes (Fig. 2D, Supplementary Fig. S4C). Tfh cell frequencies in LNs draining the immunization sites significantly increased after t.c., i.d., and s.c. immunization (*P* < 0.0001) (Fig. 2E). The absolute numbers of Tfh and Tfh Bcl6^+^ cells were also significantly increased following these routes of immunization (Supplementary Fig. S4D, E). Env-specific IgG titers in plasma were measured one week after the prime-boost schedule using Env-coated Luminex® beads. Among all immunization routes, the s.c. and i.d. routes significantly boosted Env-specific IgG titers (*P* < 0.01 and *P* < 0.001, respectively) (Fig. 2F). These results confirmed previous findings that the s.c. route induces Env IgG responses^34^, but also revealed that the i.d. route elicits stronger humoral responses, with fold changes in mean titers of 11.7 ( ± 3.3) for i.d. compared to 3.6 ( ± 1.5) for s.c.

In summary, topical and t.c. immunization routes were less effective for delivering the αLang.Env vaccine. Although GC and Env-specific GC B cells were detected after t.c. immunization, the weaker Tfh response suggests a deficiency in the GC/Tfh interaction, which is crucial for a robust humoral response. The i.d. route emerged to be effective, eliciting the highest levels of Env-specific IgG and a robust Tfh response (Fig. 2G). Notably, only in the i.d. group was Env-specific IgG significantly correlated with Tfh cell frequencies (*P* = 0.0014).

### Env-specific adaptive immune response after αLang.Env skin vaccination depends on Langerhans cells

To decipher whether the immune response detected in B6 mice following Langerin-targeting of HIV-1-gp140z described above was exclusive to targeting Langerin of LCs and/or of cDC1s, we used the *Xcr1*^DTA^ mouse model in which cDC1s are constitutively depleted by the expression of diphtheria toxin subunit A, in comparison with their littermates *Xcr1*^iCre-mTFP1^ (CTRL), in which both LCs and cDC1s are present, similar to B6 mice^38^. Each genotype of mice was assigned to separate groups, and we applied the different routes of immunization (s.c., i.d., t.c.) and the same scheme Prime-Boost skin application of αLang.Env (3 weeks of interval between shots) as described in Fig. 2A and the induced immune response was evaluated 6 days after the boost. No significant differences in Tfh, GC B cell, or PC frequencies were observed between PBS-immunized mice of the different genotypes, so these were combined as non-immunized controls (referred to as “PBS”). Background Env IgG titers measured by Luminex® (MFI) were identical across all groups of mice.

**Fig. 3:**
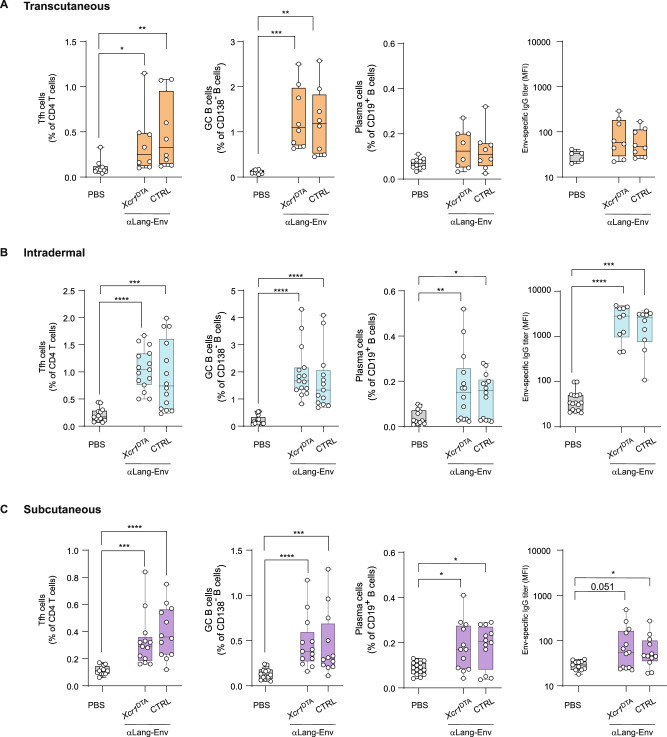
Cellular and humoral responses to αLang.Env are maintained in mice lacking Langerin^+^ dermal cDC1. From left to right: frequencies of Tfh cells among CD4^+^ T cells, GC B cells among CD138^–^ B cells, PC among total B cells and Env-specific IgG levels in serum of *Xcr1*^DTA^ versus control (CTRL, *Xcr1*^iCremTFP1^) mice immunized via (**A**) t.c. (laser P.L.E.A.S.E.), (**B**) i.d. and (**C**) s.c. routes. The frequencies of studied populations and Env IgG titers were not significantly different between non-immunized CTRL and *Xcr1*^DTA^ mice and thus were pooled as non-vaccinated (“PBS”) control group. Non-parametric Kruskal-Wallis tests with Dunn’s multiple comparison post hoc test, *P < 0.05, **P < 0.01, ***P < 0.001, ****P < 0.0001. Non-significant responses are not indicated. Data are representative of at least two experiments.

Significant increases in Tfh cells were observed in CTRL mice following immunization via t.c., i.d., and s.c. routes, with average frequencies of 0.48% ( ± 0.14), 0.93% ( ± 0.17), and 0.4% ( ± 0.05) of CD4^+^ T cells, respectively (Fig. 3, left panels). Similarly, GC B cell frequencies increased, with averages of 1.24% ( ± 0.26), 1.68% ( ± 0.31), and 0.45% ( ± 0.1) of CD138^–^ B cells, respectively. These responses were not significantly different compared to *Xcr1*^DTA^ mice. PC frequencies were significantly higher following i.d. and s.c. immunization (*P* < 0.05), with averages of 0.14% ( ± 0.02) and 0.19% ( ± 0.02) of CD19^+^ B cells, and again showed no significant differences from *Xcr1*^DTA^ mice. As an internal control, considering the established role of cDC1 in CD8^+^ T cell priming, the frequency and absolute numbers of CD62L^–^CD44^+^ CD8^+^ T effector cells induced following i.d. immunization were impaired in *Xcr1*^DTA^ lacking cDC1 (Supplementary Fig. S5A).

When evaluating Env IgG titers via Luminex®, responses to αLang.Env were significant in CTRL mice after i.d. (*P* < 0.01) and s.c. (*P* < 0.05) immunization but not after t.c. application, consistent with previous findings in B6 mice (Fig. 3, right panels). Env humoral responses were not significantly different in *Xcr1*^DTA^ mice. Notably, Env IgG responses after s.c. immunization were two logs lower compared to i.d. immunization, confirming that the i.d. route was more effective. Additionally, s.c. administration of αLang.Env in *Xcr1*^DTA^ mice induced Env IgG titers at day 27 that approached significance (*P* = 0.051) compared to PBS controls, with responses confirmed when analyzing the overall Env IgG kinetics (Supplementary Fig. S5B, C). In the absence of cDC1s, i.d. immunization of *Xcr1*^DTA^ mice with αLang.Env led to significantly higher Env-specific IgG titers in serum compared to the non-targeted condition (coh-Env) (Supplementary Fig. S5D).

Taken together, these results show that the specific targeting of langerin in absence of cDC1 in *Xcr1*^DTA^ mice neither impaired nor enhanced the GC/Tfh reaction induced by αLang.Env application. This indicates that LCs might be the primary skin cell subset responsible for priming CD4^+^ T cells and triggering Tfh cell differentiation, leading to the activation of GC reaction in dLN. Moreover, those results confirm that the i.d. route of vaccination was the most efficient for generating Env-specific humoral responses, corroborating our results on B6 mice.

## Discussion

In this study, we investigated the impact of different skin immunization routes (i.d., s.c., t.c. and topical) on T and B cell priming and subsequent humoral responses. We focused on αLang.Env, a vaccine candidate engineered to target Langerin-expressing cells, aiming to elucidate how distinct subsets of skin APCs, particularly LCs and cDC1, modulate GC responses in dLNs, Tfh cell formation, and antigen-specific antibody production. We first assessed the proximal dLNs and T cell activation following immunization using an OVA surrogate antigen and OT-I/OT-II adoptive T cell transfer assays. All skin routes were capable of inducing antigen-specific CD8⁺ (OT-I) and CD4⁺ (OT-II) T cell proliferation to varying degrees. However, the i.d. route showed superior T cell priming, particularly in the auricular lymph nodes, indicating more effective antigen delivery and presentation. The reduced immunogenicity observed with the t.c. and topical routes may reflect insufficient antigen penetration post-electroporation (t.c.) or tape stripping (topical Hilltop chamber). These findings suggest that increasing the vaccine dose may enhance delivery, uptake, and processing by epidermal and dermal APCs.

Expanding upon these findings, we evaluated the immunogenicity of αLang.Env delivered via the different skin routes. All routes increased GC B cell frequencies in dLNs. However, only the i.d., s.c., and t.c. routes led to significant increases in Env-specific GC B cells and Tfh cells. Among these, i.d. administration uniquely induced a marked increase in systemic Env-specific IgG titers, which correlated with elevated Tfh cell frequencies. These data underscore the ability of i.d. immunization to efficiently engage critical immune components, including Tfh cells and GC B cells, thereby driving strong humoral responses. Intradermal delivery targets skin layers rich in APCs, especially LCs and dermal DCs, promoting efficient antigen uptake and rapid immune activation. In contrast, s.c. injections deposit antigens in adipose tissue with fewer APCs and slower antigen drainage to LNs, which can limit immune responses, especially in the absence of adjuvants. Thus, as proposed by Romani N. and coll.^40^, the i.d. route might offer superior LC targeting, faster drainage, and better antigen presentation, that might contribute to the potency of this route. Although slightly lower Env-specific GC B cell frequencies were observed in the i.d. group compared to others, this did not compromise the antibody response. Instead, the i.d. route yielded the highest systemic antibody levels, suggesting that early Tfh / B cell interactions and rapid plasma cell differentiation may compensate for GC size.

To dissect the respective contributions of LCs and cDC1, we utilized *Xcr1*^DTA^ mice, which lack cDC1. Targeting LCs alone in these mice elicited immune responses comparable to those in wild-type or *Xcr1*^Cre-mTFP1^ control mice, where both LC and cDC1 subsets were present. Furthermore, we confirmed that i.d. administration of Env in *Xcr1*^DTA^ mice led to significantly enhanced antibody responses when the antigen was targeted to LCs, compared to the non-targeted form. These findings are consistent with previous studies highlighting the immunological advantage of LC-targeted vaccination strategies and highlight the essential role of LCs in initiating CD4⁺ T cell responses and driving GC formation upon anti-Langerin skin immunization^31,33,34,37^.

Although LCs are pivotal, other studies indicate that additional skin DC subsets, such as CD103⁺ cDC1 or cDC2, also contribute to adaptive immunity^41^. While cDC2 have traditionally been associated with humoral responses due to their advantageous positioning, ability to activate CD4^+^ T cells, and presentation of native antigen to B cells, recent studies using targeted cDC1 demonstrated that this subset can also play a role in activating naïve B cells and priming T cells^42^. cDC1s in lymphoid tissues are located near B cell follicles, facilitating their interaction for direct presentation of native antigen^43^. Moreover Krishnaswamy et al. showed that cDC1s, but not cDC2s induced Tfh-dependent antibodies response in mice^44^. In contrast, other authors describe that cDC2s located in strategic areas of secondary lymphoid organs present higher levels of CXCR5 Tfh ligand, triggering rapid CD4^+^ T cell clustering and activation in spleen and dLN after immunization^45,46^. Thus, while LCs, cDC1, and cDC2 each contribute to humoral immunity, our findings reinforce previous evidence that specifically targeting LCs represents a particularly effective strategy^33,37^. Our group has already shown that the i.p. administration of an anti-langerin.Flu HA1-1 complex enhances Flu HA1-1-specific responses, but the i.p. route is not practically transposable to human vaccination^31^. While intramuscular (i.m.) injection is standard for many vaccines, it may not be suitable for DC-targeted strategies due to the low density of cDCs and the absence of LCs in muscle tissue^47^. In contrast, our findings highlight the potential of low-dose, adjuvant-free i.d. immunization to induce robust antibody responses, supporting earlier reports demonstrating that targeting DC subsets within defined skin layers can critically influence the quality of adaptive immune responses. For instance, Terhorst et al. demonstrated that laser microporation followed by XCR1⁺ DC-targeted vaccination promoted robust T cell responses^39^. The mild inflammatory milieu created in the dermis by skin laser microporation itself most likely favored the development of potent T cell responses in the absence of exogenous adjuvants. Likewise, i.d. injection of HIV-1 Gag/p24-nanoparticles selectively engaged skin-derived DCs, including LCs, to promote Tfh cell differentiation and GC formation^48^. In our model, i.d. immunization offered the most favorable conditions, with effective LC engagement, and efficient initiation of Tfh / B cell interactions. While we observed slightly lower frequencies of Env-specific GC B cells in the i.d. group, vaccinated animals exhibited the highest systemic Env-specific IgG titers, suggesting that factors beyond GC B cell frequency, such as early and effective Tfh / B cell interactions and accelerated differentiation into plasma cells, may drive superior antibody output. This supports a growing body of evidence indicating that the timing, quality, and coordination of immune events, rather than absolute cell numbers alone, are critical determinants of vaccine efficacy^49–51^. The sequence of events spanning Tfh priming, GC B cell class-switching, plasma cell differentiation, and antibody production is not necessarily synchronous or linear. Antigen retention by follicular dendritic cells (FDCs) as well as the persistence and spatial dynamics of antigen presentation in the GC, may further influence the temporal evolution of the response^50,52^. Therefore, both the dose and biodistribution of antigen in the skin, and the timing of immune stimulation, should be considered when comparing immunization routes. Altogether, our data reinforce the potential of LC-targeting skin vaccines to qualitatively enhance humoral immunity through optimized Tfh / B cell collaboration and GC dynamics^34,35,37,39^.

To conclude, the i.d. route stands out as a promising approach among the skin-based strategies tested. These results pave the way for the rational design of next-generation DC-targeting vaccines not only for HIV-1 but also for other infectious diseases, enabling more precise control over immune activation and antibody programming. Our results focused on the magnitude of the humoral response, as assessed by anti-Env antibody levels and the frequency of germinal center Tfh and B cells, to provide a clear and direct readout. The study is still limited by the absence of inter-group comparisons, which would require a larger sample size to thoroughly assess Tfh responses and other immune parameters across the different immunization routes. While our analysis focused on anti-Env IgG levels and the GC/Tfh reaction in dLN, future studies should assess qualitative parameters such as antibody durability, neutralization capacity, and mucosal protection. Finally, our current approach did not formally exclude potential contributions from other skin-resident DC subsets, nor does it definitively establish the exclusive involvement of LCs. Further studies using models such as huLangerin transgenic mice^37^, which enable specific targeting of skin LCs with anti-human Langerin mAb will be essential to clarify the precise role of LCs. However, we showed that in the absence of cDC1s, αLang.Env led to significantly higher Env-specific IgG than Env alone. This underscores that non-specific uptake by other DC subsets is not sufficient to generate equivalent antigen-specific humoral responses observed with anti-Langerin targeting.

## Methods

### Vaccine constructs and formulations

The αLang.Env vaccine constructs have been designed and produced as previously published^34^. Briefly, the anti-Langerin mAb was developed in-house (clone 4C7^31^, GenBank sequences JX002668, JX002669) and exhibited cross-binding to both mouse and human Langerin receptors. These antibodies featured a mouse IgG2b constant region and had a dockerin (doc) domain (residues 551-625 of GenPept Sequence ID: P0C2S4.1) fused to the C-terminal end of the H-Chain^31^. αLang 4C7.doc was produced in stably transfected Expi-CHO-S cells (Thermo Fisher), captured from culture supernatant on protein A affinity matrix and purified by FPLC (Akta, GE Healthcare). Batches of αLang.doc were further purified by reverse chromatography using a SEC 26/600 column (Cytiva), to avoid any aggregates or degraded product (see Supplementary Fig S2). The HIV-1 Envelope Clade C 96ZM651 gp140 sequence (GenBank accession # AY181197.1 residues 94 to 2064) was inserted at the C-terminus of a Cohesin (Coh) domain (residues 150-169 appended to residues 165–314 of GenPept Sequence ID: CAA47806.1) via a flexV1 flexible linker spacer (residues 468–494 of GenPept Sequence ID: AJD85777.1)^31^ and produced independently, captured on EPEA C-tag column and purified by FPLC. All recombinant mAbs were stored in 1 M Arginine 100 mM Tris-HCl (pH 7; 4 mg/mL; −80 °C) and dialyzed into Dulbecco’s phosphate buffered saline (DPBS) before immunization. Quality controls were done by SDS-PAGE followed by Coomassie staining. All reagents and final products utilized in this study tested less than 0.5 ng lipopolysaccharide per milligram of protein (endotoxin kit, ThermoFisher). His-tagged trimeric HIV-1 Envelope proteins were produced in stably transfected Expi-CHO-S cells, purified on a HIS-capture column (ThermoFisher) and then biotinylated according to manufacturer’s instructions (Avidity, US).

The αLang.Env was prepared by an equimolar mix of Coh antigen and the doc vehicle, 1 h at 37 °C in DPBS containing calcium and magnesium. The OVA.CT.Alum formulation consisting of 10 µg per dose of ovalbumin imject^™^ (Thermo Fisher Scientific), adjuvanted with Cholera Toxin (Sigma Aldrich) (0.1 µg/dose) adsorbed in Thermo Scientific Imject^™^ Alum Adjuvant (200 µg/dose) was prepared in sterile DPBS 1x (Sigma-Aldrich) 24 hours prior to immunizations.

### Mice

*Xcr1*^Cre-mTFP1^ mice were generated as previously described^38^ and were crossbred with *Rosa26*^LSL-DTA^ [B6.129P2-*Gt(ROSA)26Sor*^tm1(DTA)Lky/J^] mice^53^, generating heterozygous *Xcr1*^DTA^ mice where Cre-inducible deletion of the floxed-STOP cassette allows for the expression of diphtheria toxin subunit alpha gene (DTA*G128D) and the toxin produced in Xcr1-expressing cells causes a general ablation of cDC1 in those mice. OT-I^54^ and OT-II^55^ mice were kept on a CD45.1(Ly5.1)/CD45.2(Ly5.2) B6 background and were employed as donors in adoptive transfer and proliferation assays. C57BL/6^J^ (B6) mice were provided by Charles River. All mice were maintained on C57BL/6^J^ (B6) background and in a specific pathogen-free environment. All in vivo procedures were conducted in adherence to protocols approved by the Ethics Committee of Marseille (approval APAFIS number 28232-2021020512136337) in compliance with institutional, national, and European guidelines governing the ethical treatment and care of animals used in research.

### Adoptive transfer of CTV-labelled OT-I and OT-II T-cells

Superficial lymph nodes (auricular, cervical, brachial, axillary and inguinal LN), mesenteric LN and spleen of transgenic OT-I or OT-II mice were harvested, and total cells suspensions were prepared by smashing the organs through a 70 µm nylon mesh. Single-cell suspensions were then washed with sterile PBS and spleen cells were subjected to red blood cells lysis using Red Blood Cell Lysis Buffer (eBioscience). OT-I and OT-II cells were isolated by negative selection and purified using CD8^+^ or CD4^+^ T cell enrichment Kits (Dynabeads Untouched mouse CD8^+^ or CD4^+^ T Cells, ThermoFisher Scientifics) according to the manufacturer’s guidelines. Purified T cells were subsequently counted and labelled with 5 µM CellTrace Violet (CTV; Molecular Probes) for 30 minutes at 37 °C, following the manufacturer’s instructions. CTV labelled OT-I or OT-II T cells were washed and resuspended in sterile DPBS at a concentration of 1.5 × 10^7^ cells/mL, and 200 μl (3 × 10^6^ cells) and were adoptively transferred into B6 mice by intravenous injection, as per standard procedures^56–58^.

### Immunizations and study design

Between *n* = 3–5 mice (8–12 weeks old) were sorted into different groups comprising different skin routes of immunization, and each experiment was repeated 2–3 times. Four different routes and sites of application were compared: i. subcutaneous (s.c.), to access hypodermis at the axillary flank of mice; ii. intradermal (i.d.) to access preferably dermis of the dorsal side of the ear pinna skin; iii. transcutaneous (t.c.) to access both the epidermis and dermis of the dorsal side of ear pinna skin, and iv. topical route to access preferably the epidermis of the lower ventral abdomen skin. Prior to all procedures, mice underwent anesthesia via an intraperitoneal injection containing zolazepam hydrochloride (Zoletil 20, Laboratoires Virbac, France) and 6 mg/kg of tiletamine along with 3 mg/kg of xylazine (Xilor 2%, Bio 98 Srl, Italy). Both s.c. and i.d. injections were done using 0.3 mL 30 G insulin syringes (BD Micro-Fine+ Demi 0.3 mm x 8 mm). For the t.c. groups, prior to vaccine application, the dorsal side of the ear pinna skin was electroporated using the P.L.E.A.S.E. device (Pantec Biosolutions)^59^ and vaccines were topically applied on the electroporated skin. P.L.E.A.S.E. configurations were set as follows: pulse duration: 75 microseconds, number of pulses per pore: 2, pore array size: 14 square millimeters, pore density: 8 percent, fluence: 11.9 Joules per square centimeter and RepRate: 200 Hertz, as described previously^39^. The Hilltop chamber system (Cliantha Research) was used for topical application. Mice were shaved in the lower ventral surface of abdomen, the skin was tape-stripped 25 times (Scotch 3 M Magic #810, 19 mm×33 mm) and the vaccine, embed in the cotton pad of the Hilltop, was applied onto the treated skin and left in contact for 24 hours. All groups received the same dose of vaccines (20 µg of αLang.Env or 10 µg of OVA) but the volumes were adjusted for each route of immunization. The volumes of injection for s.c., i.d. and t.c. application were equally divided between both right and left sides of axillary flank (50 µL each for s.c.) and the skin of the dorsal side of both ear pinna (10 µl each for i.d. and t.c.), respectively. The volume of vaccine used in the Hilltop device was 100 µL (embedded in the device cotton pad) applied at one site of mice abdomen skin. Mock groups, injected with the same volume of sterile DPBS at each site of immunization, were used as Negative Control in all experiments. Two different immunization schemes were used depending on the goal of each experiment and the antigen used, either OVA or αLang.Env. Twenty-four hours after adoptive transfer of CTV-labelled OT-I or OT-II T cells, B6 mice were immunized with OVA.CT.Alum formulations through the aforementioned skin routes (s.c., i.d., t.c. and topical). 72 h after immunization (day 3) skin-dLNs (auricular, brachial, axillary and inguinal) were harvested for each mouse and filtered through a 70 μm cell strainer. Cells were then rinsed with sterile DPBS and stained to follow OT-I and OT-II proliferation by FACS (Fig. 1A; Sup. Fig. S1). B6, *Xcr1*^Cre-mTFP1^ and *Xcr1*^DTA^ were primed at day 0 with the HIV-1-gp140z targeting Langerin (αLang.Env) through the aforementioned skin routes (s.c., i.d., t.c. and topical). Three weeks later (day 21) mice were boosted with the same dose of the vaccine candidate. Around one week after the boost (day 27) mice were sacrificed and samples (blood and dLN) were collected for immunophenotyping of lymphocytes (GC, Tfh and T memory cells) as well as for measurement of Env-specific IgG levels in plasma (Fig. 2, Sup. Fig. 3).

### Cell extraction and preparation

After each protocol of immunization, mice were sacrificed via a CO_2_ chamber and samples including blood and lymph nodes (LN) were harvested. Total cells suspensions were prepared by smashing the LN through a 70 µm nylon mesh. Cells were washed, resuspended in DPBS/2% FCS (5 mM EDTA), counted and adjusted for 3 × 10^6^/mL for FACS staining. Approximately 100 μl of fresh blood was collected on EDTA capillary tubes via the ocular plexus of isoflurane-anesthetized animal, then RBC lysis was performed for 10 minutes and the PBMC were washed twice with DPBS/2% FCS and then stained for FACS analysis.

### FACS cellular immunophenotyping

Prior to staining, lymph nodes and blood single cell suspensions were treated Fc block (clone 2.4G2) (Thermo-Fisher) in FACS buffer (DPBS at 2% of FCS and 5 mM of EDTA) at 4 °C for 15 minutes to prevent non-specific binding via Fc receptors. Cells were washed with DPBS and stained for viability with Zombie UV dye (BioLegend) at 4 °C in DPBS for 20 min. Cells were washed, and incubated for 30 min at 4 °C with in-house biotinylated trimeric HIV-1-gp140z^34^. After washes, cells were stained for surface markers. Fluorochrome-conjugated mouse antibodies CD3 (145-2C11 or 17AZ), CD4 (GK1.5 or H129.19), CD5 (53-7.3), CD8b (H35-17.2), CD11b (MI/70), CD19 (1D3), CD38 (90/CD38 Ab90), CD44 (IM7), CD45.1 (A20), CD45.2 (104), CD62L (MEL-14), CD95 or FAS (29 F.1A12), CD138 (281-2), CD161.1c (PK136), CD279 or PD-1 (11/41), CXCR5 (2G8), GL7 (GL7), IgD (11-26c2a), IgG (Poly4053) (BioLegend), IgM (R6-60.2), MHCII (M5/114.15.2) were used for surface staining. Anti-Biotin-APC (REA76, Miltenyi Biotec) and anti-Biotin-PE (104-C5, Miltenyi Biotec) were used to detect the binding of the biotinylated trimeric HIV-1-gp140z to specific B cells. For CXCR5 staining, cells were pre-incubated with the antibody for 30 min at 4 °C in PBS/2% FCS. The remaining antibodies were mixed in FACS buffer, added following this step, and incubated at the same conditions for further 30 min. Following extra-cellular staining, cells were fixed with CytoFix solution from the Kit mouse/rat FoxP3 (eBioscience) at room temperature for 40 minutes and stained for intracellular marker Bcl6 (K112-91) diluted in CytoPerm solution for 40 minutes and subsequently rinsed and resuspended in FACS buffer. Unless specified, all antibodies were procured from BD Biosciences. 1 × 10^4^ Truecount absolute counting beads (BD) were added to the tubes before FACS acquisition allowing accurate quantification of the cells. Fluorescence was analyzed using a FACS Fortessa or FACS Symphony flow cytometers (BD Bioscience) and FlowJo software. The gating strategies for OT-I and OT-II T cell proliferation are described in Supplementary Fig. S1 and for GC B, Tfh and T memory cells in dLN and blood are described in Supplementary Fig. S3.

### Plasma preparation

Blood was collected and allowed to coagulate for one hour, followed by centrifugation at 1000 x g to isolate plasma. Samples were frozen at −80 °C until analysis of humoral responses.

### Humoral response measurements

Plasma levels of HIV-1-specific IgG were assessed using a specialized multivariate Luminex® assay, as detailed in prior descriptions^34^. MagPlex beads (Bio-Rad, France), were activated by rinsing with DPBS and subsequently incubated with 6.3 μg of HIV-1 Env gp140z antigen, produced in-house, at 4 °C overnight with agitation, following the manufacturer’s guidelines. Beads bound to Env antigen were washed with DPBS, suspended in blocking buffer, and finally in 150 μl of storage buffer, counted using an Auto-2000 cell counter (Nexcelom) and stored at 4 °C in the dark until use. At the day of the experiment, the prepared beads were diluted to a concentration of 50,000 beads/mL in PBS, and 50 μl of this solution was dispensed into Bio-Plex Pro 96-well Flat Bottom Plates (Bio-Rad). After two washes with 0.05% tween PBS using a magnetic plate washer (Bio-Rad), 50 μl of individual serum samples diluted at 1:100 in DPBS were added to each well. Env-specific IgG of mice plasma beads binding occurred at room temperature for 30 minutes with agitation, followed by the addition of an anti-mouse IgG-PE secondary antibody (45 minutes at 0.5 μg/mL, ThermoFisher). The beads, suspended in 80 μl of Sheath fluid (Bio-Rad), were agitated for 5 minutes at 700 rpm on a plate shaker before being directly read on a Bioplex-200 plate reader (Bio-Rad) with an acquisition volume of 50 μl and DD gate settings of 5000–25,000. Median fluorescence intensities (MFI) were extracted using Bioplex Manager 6.1 software.

### Statistical analysis

Normal distribution of the data was assessed using the Anderson-Darling test, D’Agostino and Pearson test, and Shapiro-Wilk test. Statistical analyses were conducted using either one-way ANOVA with Tukey’s post-test for normally distributed data or the Kruskal-Wallis non-parametric test with Dunn’s post-test. For two-groups analysis, non-parametric Mann-Whitney t-tests were used. A significance level of *P* < 0.05 was employed for determining statistical significance. Graphs and statistical analyses were generated using GraphPad Prism software (version 10.3). Figures were assembled using Adobe Illustrator (version 29.51), and schematic representations of mouse experiments were created with BioRender. Protein structures and the interaction between αLang.doc and Coh.Env were modeled using the AlphaFold3 software^60^.

## Supplementary information

Sup Figs. 1 to 6

## Data Availability

All raw data are available on Figshare: 10.6084/m9.figshare.29236826.v1. Further related information could be accessed from the corresponding authors upon request.
