# Supplementary material for: Targeting Langerhans cells via skin delivery of HIV Envelope enhances the antibody response to vaccination
Source: NPJ Vaccines. 2025 Jul 25;10:170. doi: 10.1038/s41541-025-01214-w (PMC12297313; doi:10.1038/s41541-025-01214-w)
Supplement: Supplementary file 1 — Sup Figs. 1 to 6 [file 41541_2025_1214_MOESM1_ESM.pdf]

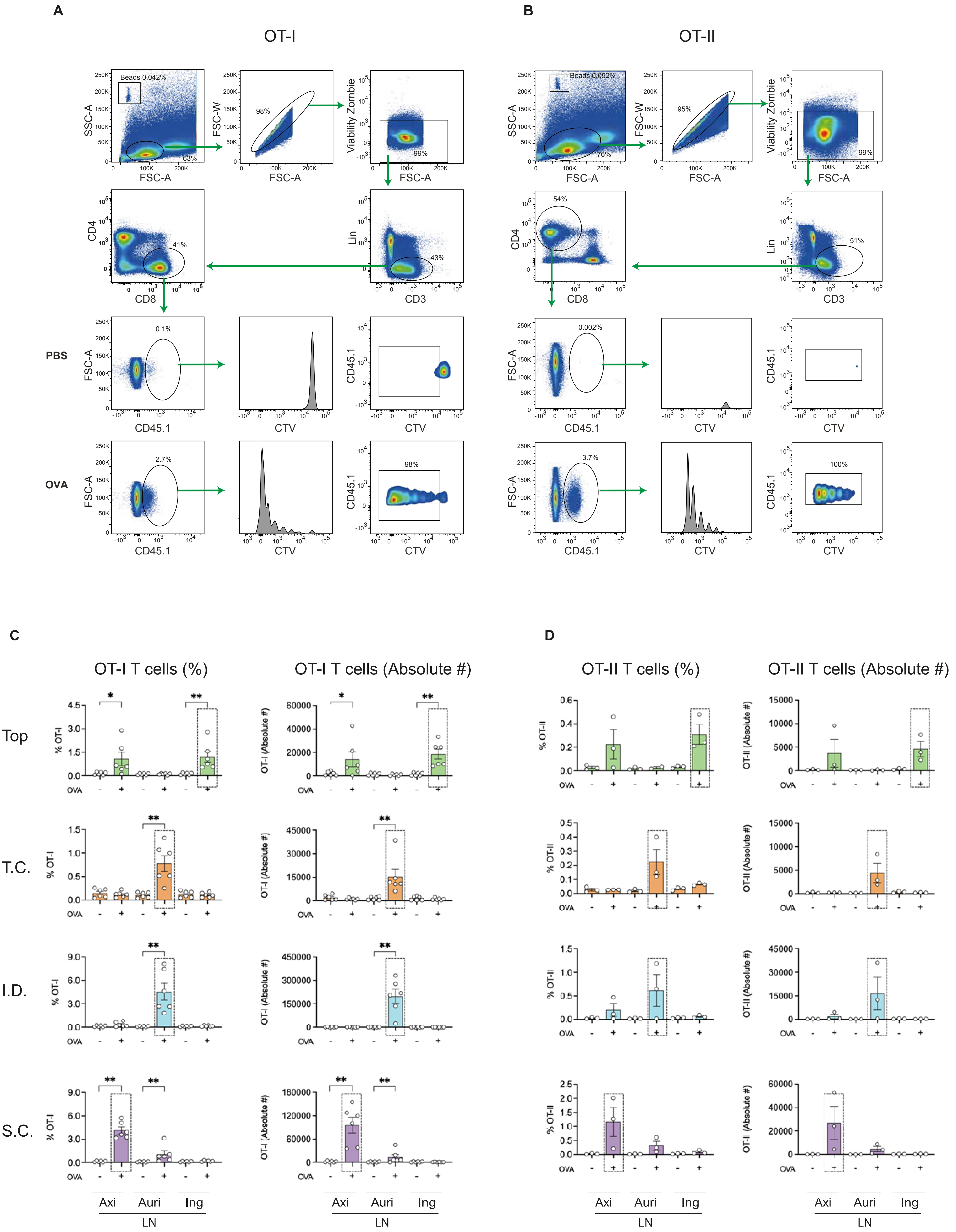


**Supplementary Figure S1: (A) OT-I and (B) OT-II T cell gating strategy in skin dLNs.** Cells were pre-gated on their size (FSA-A, SSC-A and FSC-W) and viability (Zombie–), gated as CD3+ and Lin– (the Lin channel contains CD19, CD161 and CD11b to remove respectively B lymphocytes, NK cells and myeloid cells), then CD8+ T lymphocytes (left panels) and CD4+ T lymphocytes (right panels) were selected. CD45.1+ OT-I and CD45.1+ OT-II T cells were gated respectively from CD8+ T lymphocytes (left panels) and CD4+ T lymphocytes (right panels). Their proliferation was assessed using the cell trace violet staining (CTV). Representative dot plots of non-immunized (PBS) versus OVA-immunized (with Alum) B6 mice are presented. (C) Frequencies (left) and absolute numbers (right) of OT-I and (D) OT-II T cells in respective dLNs (Axi, axillary-brachial; Auri, auricular; Ing, inguinal), after top. (green), t.c. (orange), i.d. (cyan) and s.c. (purple) immunization with OVA/alum (+) or PBS (-). For each LN, non-parametric unpaired t tests (as in Figure 1C), *P < 0.05, **P < 0.01, ***P < 0.001. Non-significant responses are not indicated. For each route of immunization, selected dLN have been squared. Data are representative of at least two experiments.

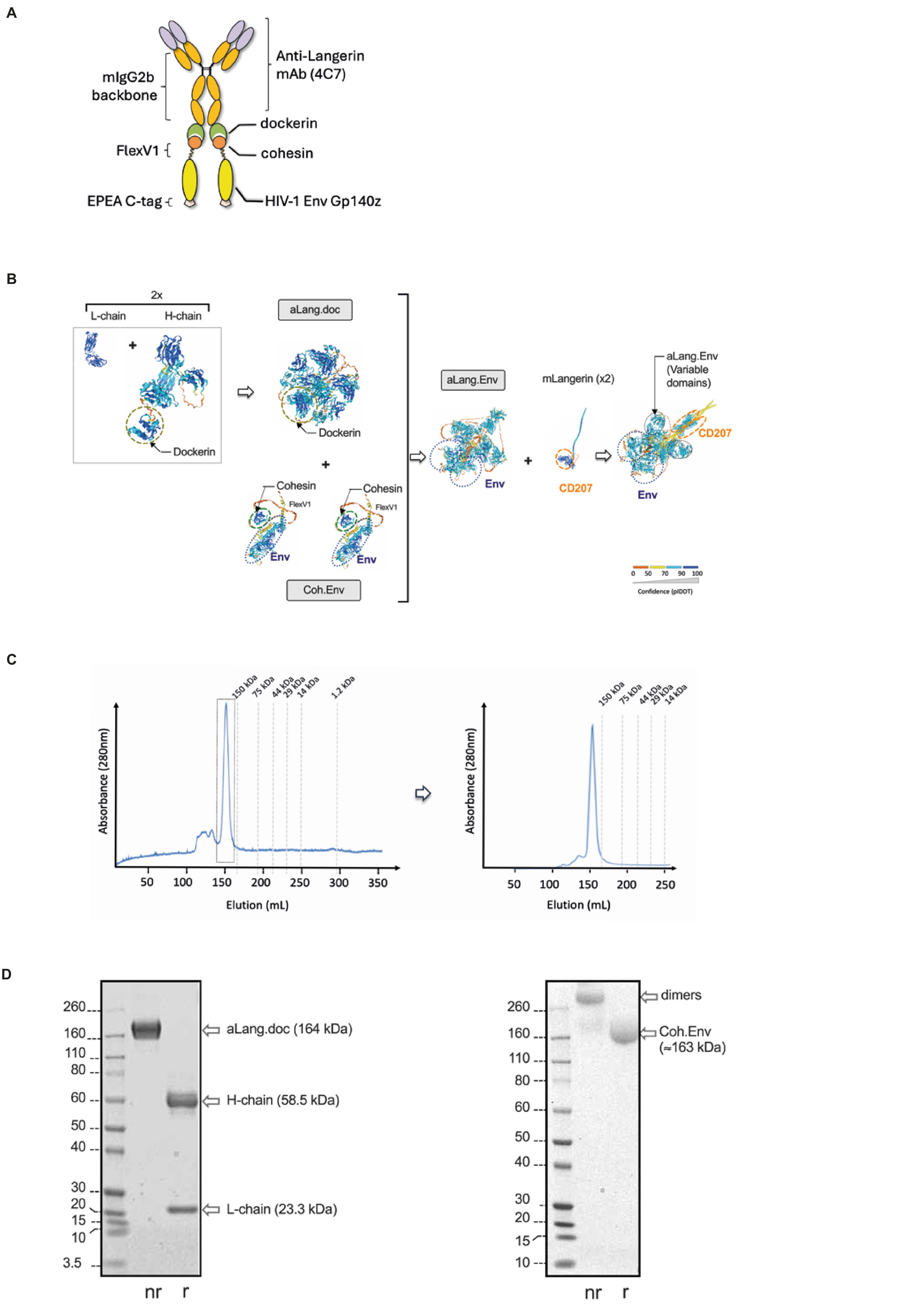


**Supplementary Figure S2: Production and quality control of the  Lang.Env vaccine. (A)** Diagram illustrating the αLang.Env vaccine. The mouse IgG2b 4C7 anti-Langerin mAb was fused to the dockerin (doc) domain at the C-terminal end of the H-chain, while the gp140z Env antigen was fused to the cohesin (Coh) domain through a flexible linker (FlexV1). A EPEA Ct-tag was present to purify the Coh-Env construct. **(B)** The anti-Langerin (Lang).doc mAb and Coh.Env constructs were combined in equimolar conditions. Their structures and the association between αLang.doc and Coh.Env were modeled using AlphaFold3 software. The dockerin was identified at the C-terminal end of the heavy chain of αLang.doc, while the cohesin domain was located at the N-terminal end of the Coh.Env construct. The Coh-Env dimers ("x2") appear to non-covalently bind to the dockerin domains, while the variable domains of αLang.Env associate with mouse Langerin receptors (right panel). The structural modeling confidence is represented by color gradients in the structural image (pLDT, per-residue model confidence score). **(C)** Before being associated with the Coh.Env, the αLang.doc mAb was purified on SEC 26/600 (left panel, dotted square) to discard any aggregates or degraded antibody. Purified Elution pic (blue line) with standard molecular weights is indicated. **(D)** SDS-PAGE with Coomassie staining of the αLang.doc mAb (left) and Coh.Env (right) under non-reduced (nr) versus reduced (r) conditions. Molecular weights (kDa) of the bands are indicated.

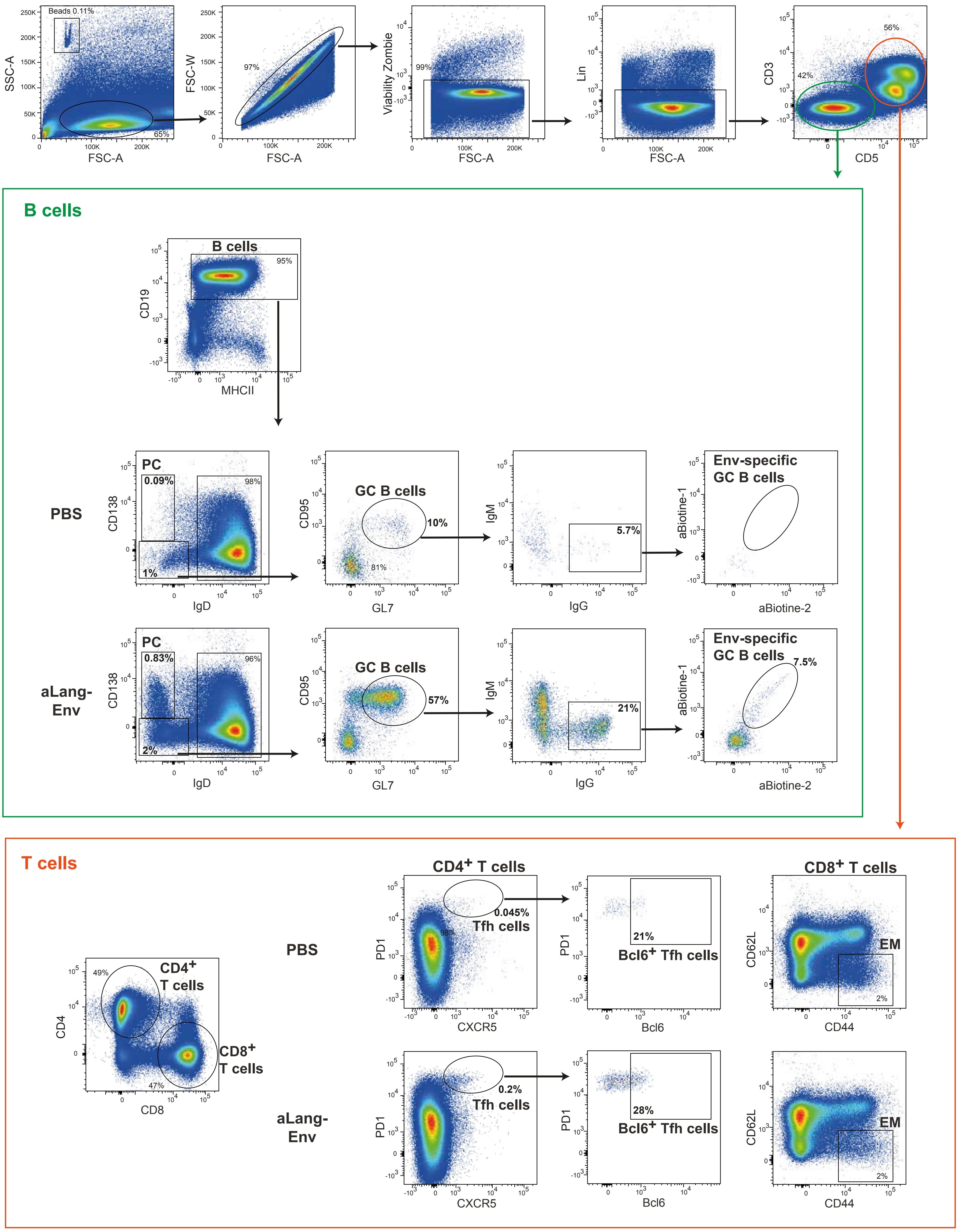


**Supplementary Figure S3: Gating strategy of the immune response induced in lymph nodes post vaccination.** Cells were pre-gated on their size (FSA-A, SSC-A and FSC-W) and viability (Zombie-) then cells were gated as Lin– (CD161 and CD11b) to remove respectively NK cells and myeloid cells, then T cells were pre-gated as CD5+CD3+ and further split as CD8+ and CD4+ T lymphocytes (**red square)**. CD4+ Tfh cells were gated as CXCR5+PD1+ and further gated as Bcl6+. Effector memory CD8+ T cells were gated as CD62L–CD44+. **(green square**) B cells were pre-gated as CD5–CD3– then further gated as CD19+MHCII+. Plasma cells were gated as CD138+IgD–. From the IgD–CD138– gate, germinal center (GC) B cells were selected as GL7+CD95+ and Env-specific GC B cells were selected as IgM– IgG+ and binding to a biotinylated trimeric Env (gp140z) produced in-house and detected with 2 anti-biotin fluorescent antibodies (population designed as "Env+/+"). Mature B cells were gated as CD38+ from GL7–CD95–. Shown here is a representative example of auricular LN after t.c. immunization with PBS or αLang-Env.

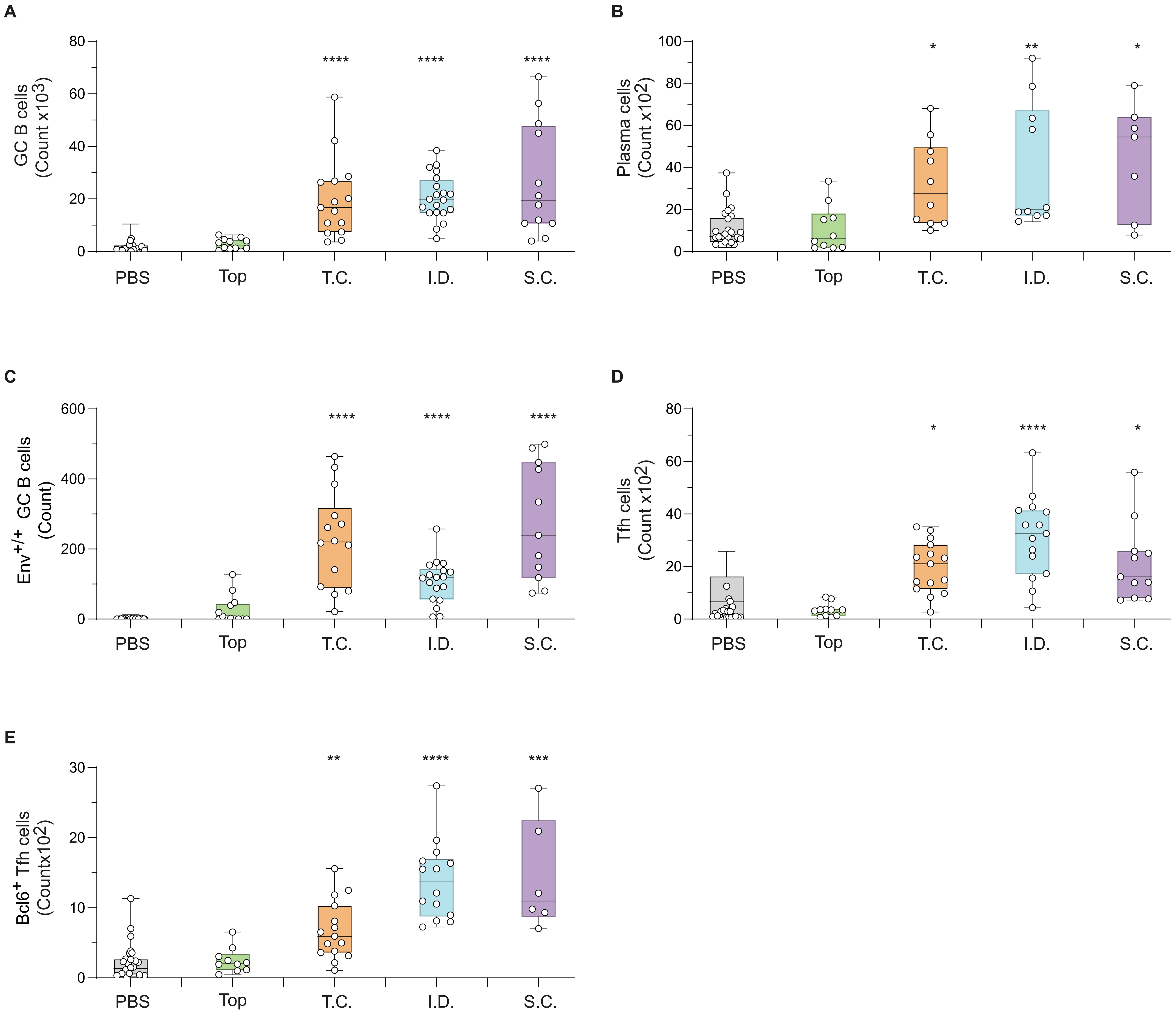


**Supplementary Figure S4: αLang.Env induces GC/Tfh reaction in C57BL/6 mice via different routes of skin immunization**. As in Figure 2, absolute numbers of **(A)** GC B cells, **(B)** PC, (**C**) Env-specific ("Env+/+") GC B cells, **(D)** PD1high CXCR5+ Tfh cells and **(E)** Bcl6+ Tfh cells in respective LNs draining each site of immunization; top. (green); t.c. (orange); i.d. (cyan) and s.c. (purple). Non-parametric Kruskal-Wallis tests with Dunn's multiple comparison post hoc test, *P < 0.05; **P < 0.01, ***P < 0.001, ****P < 0.0001. Non-significant responses are not indicated. Data are representative of at least two experiments.

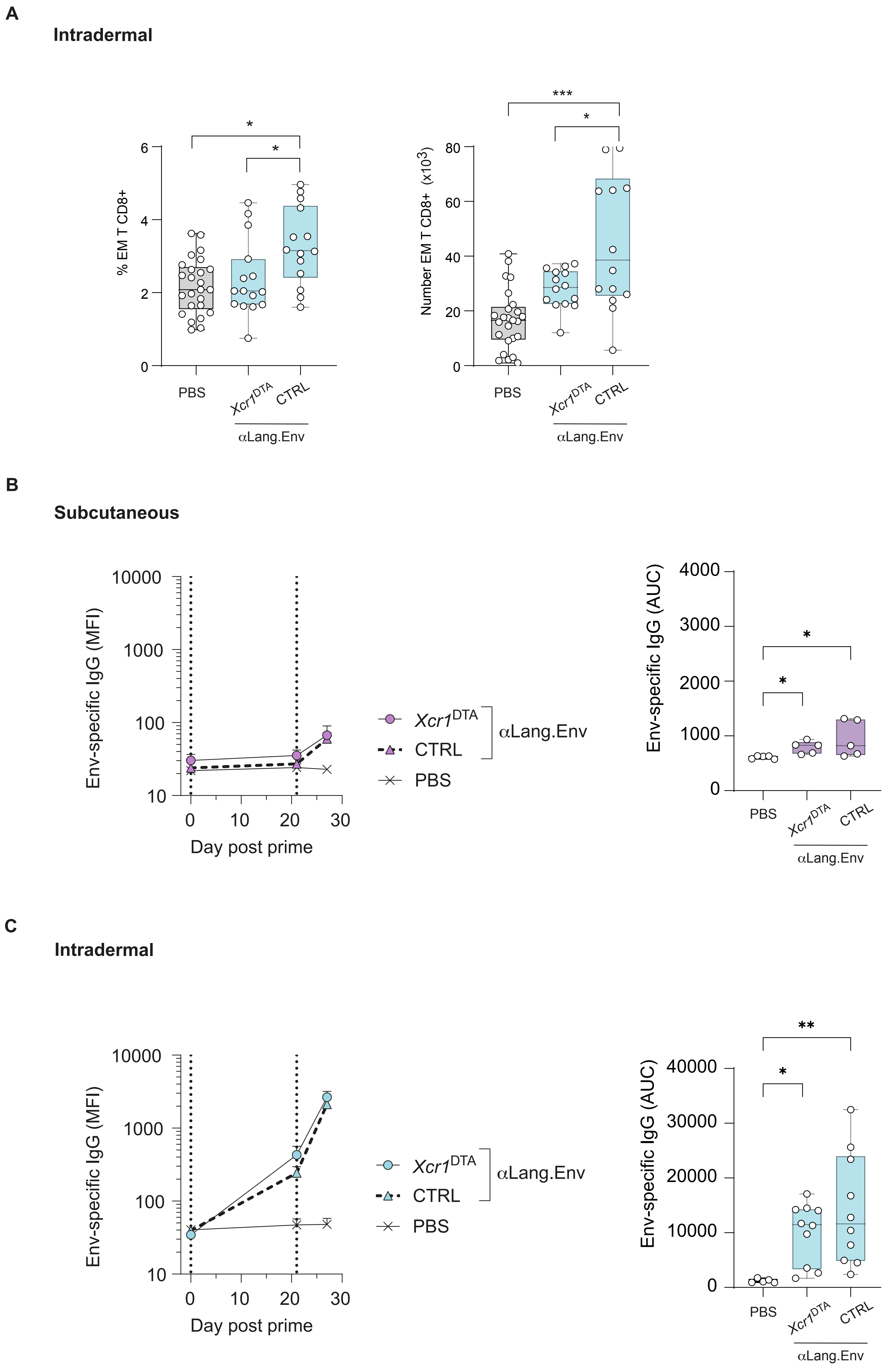


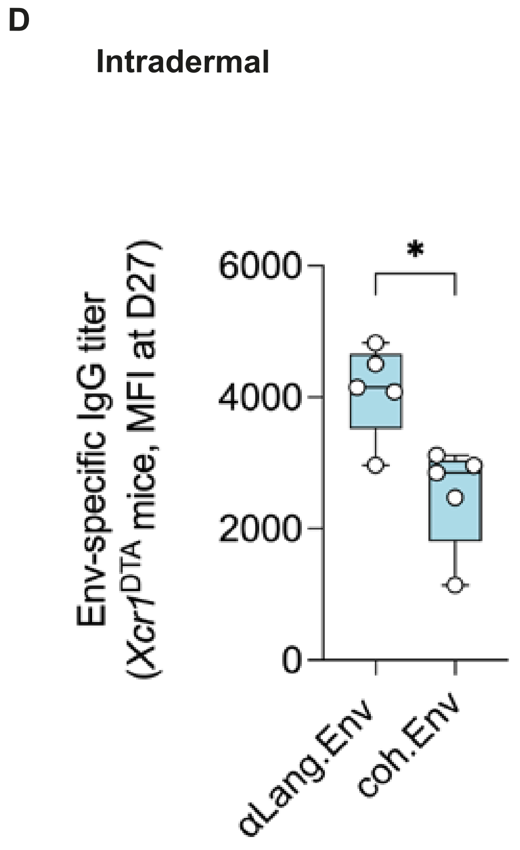


**Supplementary Figure S5: Similar induction kinetics of serum Env-specific IgG after s.c. and i.d. immunization with αLang.Env in CTRL and Xcr1DTA mice. (A)** Frequencies (left) of effector memory (EM) CD44+ CD62L– T cells among CD8+ T cells (gating shown on Supplementary Figure S3) and (right) absolute numbers in dLN of Xcr1DTA and Xcr1iCre-mTFP1 (CTRL) mice vaccinated intradermally with αLang.Env. Data are representative of at least two experiments. **(B-C)** As shown in Figures 3B and 3C, Env IgG levels were measured using Luminex at the time of the prime (Day 0), boost (Day 21), and the end of the study (Day 27) in vaccinated Xcr1DTA (solid circles) and CTRL littermate mouse models (solid triangles, dotted lines) for both (B) the s.c. (purple) and **(C)** the i.d. (cyan) routes of immunization. Non-immunized mice of both genotypes (PBS, cross marks) were included as controls. (left) Mean values for 6 mice per group (±SEM) are shown. Dotted lines indicate the immunization days. (right) Env IgG titers from Day 0 to 27 are shown as the area under the curve (AUC) for each group. Non-parametric Kruskal-Wallis tests with Dunn's multiple comparison post hoc test, *P < 0.05, **P < 0.01, ***P < 0.001. Non-significant responses are not indicated. **(D)** Env-specific IgG levels were measured post-boost in Xrc1DTA mice immunized either with αLang.Env or the non-targeted Env antigen (coh.Env). Groups of animals were compared using non-parametric Mann-Whitney unpaired t-tests; *P < 0.05. Data are representative of at least two experiments.

**A**

vPAB33
MW 1 2

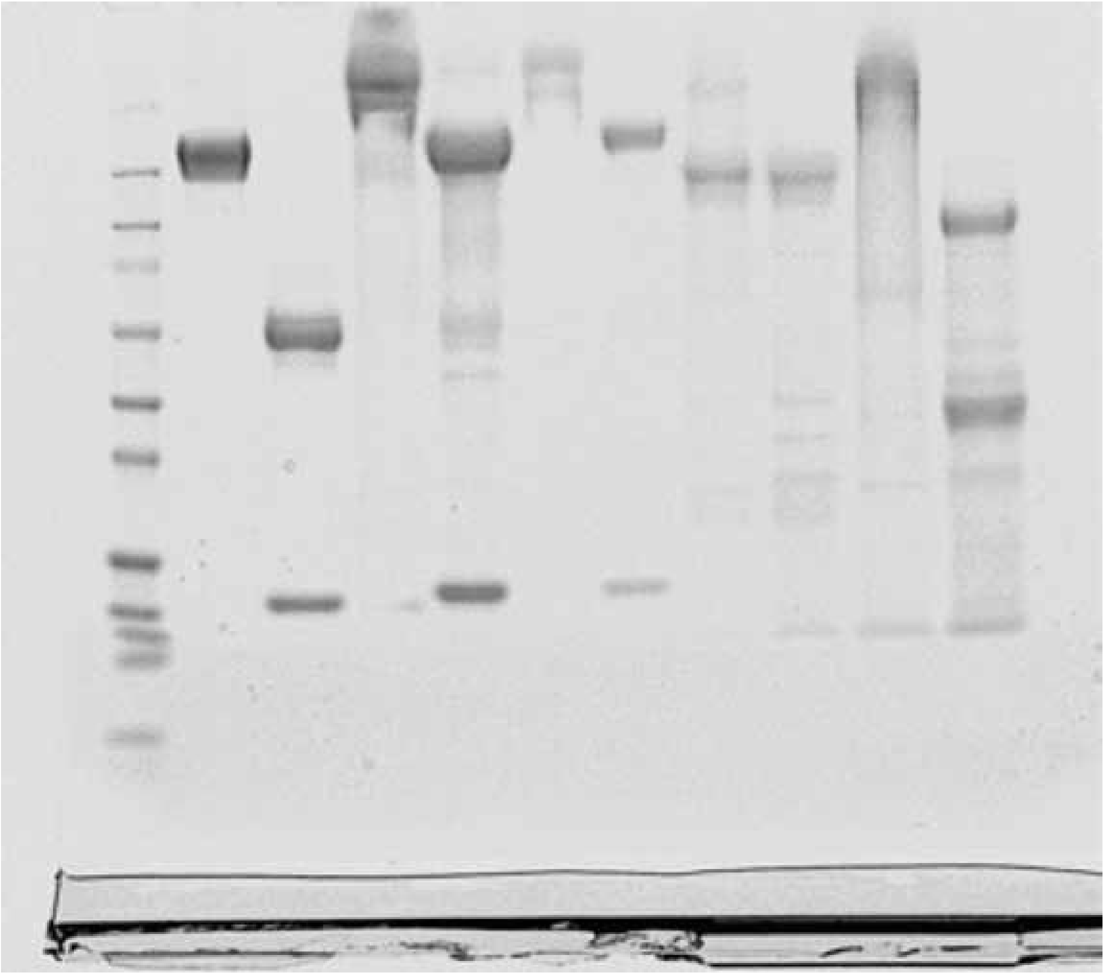

**B**

vP17
MW 1 2

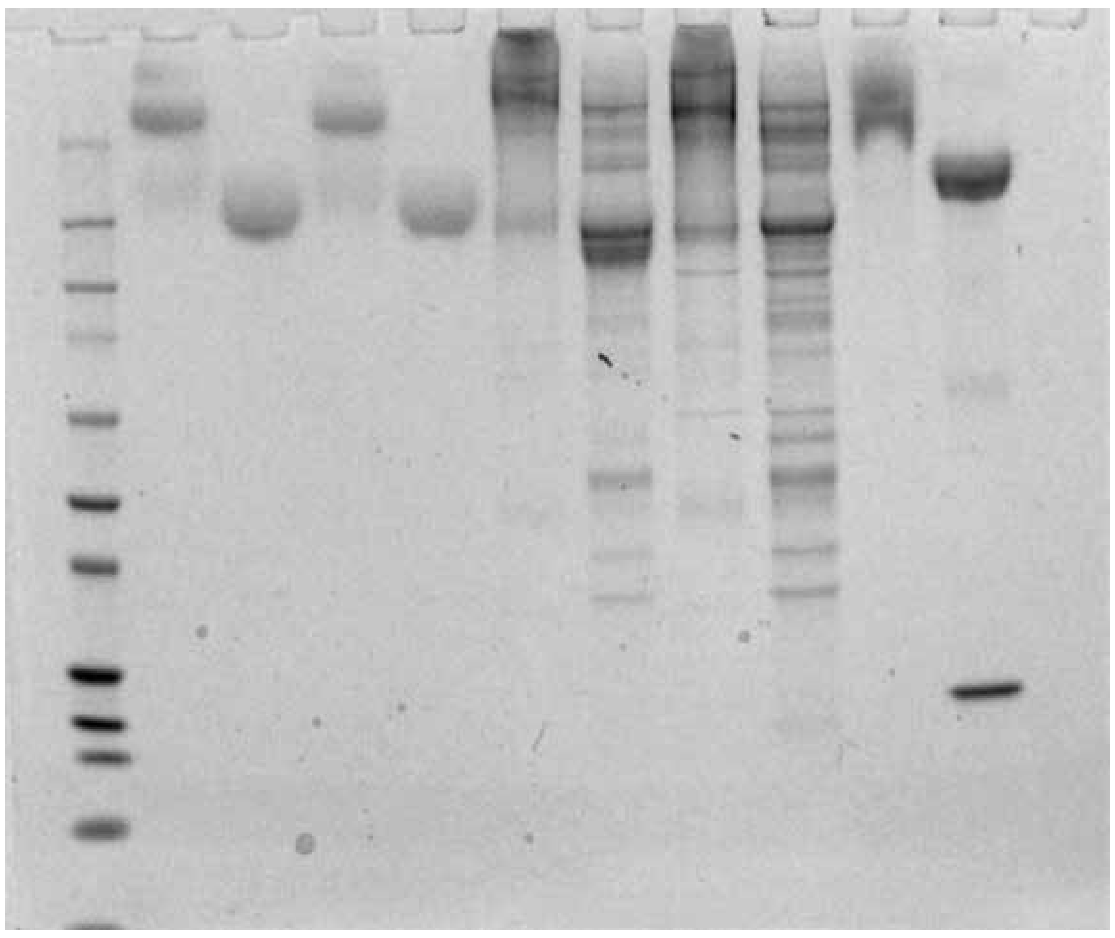

**Supplementary Figure S6: Original pictures of SDS-PAGE with Coomassie staining. (A)** aLang.doc (vPAB33) mAb as shown in FigS2D (left), and (B) Coh-Env (vP17) as shown in Fig S2D (right).
Lane 1: non-reduced, lane 2: reduced condition. Molecular weight (MW) are shown on left lanes.
